# Antimicrobial additives in resin-based endodontic sealers: a scoping review of antimicrobial efficacy, physicochemical properties, and cytotoxicity

**DOI:** 10.3389/fdmed.2026.1841705

**Published:** 2026-07-14

**Authors:** Faisal Alharamlah, Maha I. AlGhannam, Wejdan Almutairi, Faisal Alonaizan, Theeb A. Alquria, Mary Anne S. Melo, Abdulrahman A. Balhaddad

**Affiliations:** 1 College of Dentistry, Imam Abdulrahman Bin Faisal University, Dammam, Saudi Arabia; 2 Department of Dental Education, College of Dentistry, Imam Abdulrahman Bin Faisal University, Dammam, Saudi Arabia; 3 Department of Restorative Dental Sciences, College of Dentistry, Imam Abdulrahman Bin Faisal University, Dammam, Saudi Arabia; 4 Department of Comprehensive Dentistry, University of Maryland School of Dentistry, Baltimore, MD, United States

**Keywords:** bacteria, biofilms, dentistry, enterococcus faecalis, nanoparticles

## Abstract

**Introduction:**

Root canal sealers based on polymeric resins have incorporated nanostructured and antimicrobial ingredients to enhance antimicrobial activity and prevent root canal reinfection. This scoping review aimed to evaluate antimicrobial additives incorporated into resin-based endodontic sealers and their effects on antibacterial efficacy, physicochemical properties, and cytotoxicity.

**Methods:**

Electronic searches were conducted in Cochrane Library, PubMed, MEDLINE, Scopus, and Web of Science using text keywords related to resin-based endodontic sealers, antibacterial additives, physicochemical properties, and cytotoxic effects. From 1,473 records retrieved, 63 articles were eligible for full-text screening and 33 studies were included.

**Results:**

The included studies evaluated various antibacterial additives, including nanoparticles, chlorhexidine, quaternary ammonium compounds, herbal extracts, and several chemical agents incorporated into resin-based endodontic sealers. Many studies reported enhanced antibacterial activity against Enterococcus faecalis, with some demonstrating reduced biofilm formation and sustained antimicrobial effects over time. The physicochemical properties, including flow, solubility, and setting time, were generally maintained within acceptable limits. Cytotoxicity outcomes were either reduced or unchanged in most combinations, indicating favorable biocompatibility.

**Conclusion:**

In conclusion, incorporating antimicrobial additives into resin-based endodontic sealers may enhance antibacterial efficacy without significantly compromising physicochemical properties or cytocompatibility. However, further studies are required to confirm long-term biological performance and clinical effectiveness.

**Systematic Review Registration:**

https://www.crd.york.ac.uk/PROSPERO/view/CRD420261388075, identifier CRD420261388075

## 1 Introduction

Bacterial infection is among the primary etiological factors in the development of dental pulp necrosis and the subsequent periapical lesions. Chronic apical periodontitis, a common endodontic pathology that results from pulp necrosis, is characterized by pathological alterations in the periapical tissues and extensive bacterial colonization within the root canal system (1). Root canal treatment aims to attain optimal microbial reduction and eliminate inflammation within the pulpal and periapical tissues (2). Disinfection is achieved through mechanical devices, irrigation, and intracanal drugs (3). However, residual or recurrent infection continues to be the primary reason for root canal therapy failure. Nonsurgical or surgical Retreatment poses additional challenges, including reduced success rates attributed to compromised dentin structure and persistent microbial resistance (4, 5).

For successful root canal therapy, achieving a three-dimensional seal following the chemo-mechanical disinfection is essential. The ideal root canal sealer should exhibit adhesion to canal walls, size stability, oral fluids insolubility, and sufficient flowability. Adequate flow is critical for enabling the sealer to penetrate anatomical irregularities, such as fins, isthmuses, and canal sides, which contribute to the establishment of an effective seal (6). Furthermore, sealers with antimicrobial properties may help eradicate bacteria residing in such inaccessible regions. It is desirable for sealers to possess bactericidal activity or, at the very least, not support microbial growth (6).

Some evidence suggests the use of antibacterial sealers as an approach to reduce residual microbes that were not removed by chemo-mechanical instrumentation or intera-canal medicaments (7). In addition, they may serve to block microbial infiltration from the oral environment, thus delaying or preventing recontamination of the root canal system (8). When they are used in conjunction with irrigating solutions, root canal sealers that exhibit strong antibacterial activity may improve the primary endodontic treatment efficacy and decrease the risk of reinfection (9). Owing to their intimate contact with root dentin and the ability to flow into microscopic crevices, root canal sealers are well-positioned to act as a defensive barrier against persistent or recurring infections (10). Therefore, their antibacterial efficacy is a critical aspect of long-term treatment success.

However, the antibacterial effect of most conventional root canal sealers tends to diminish after setting (11). A promising solution to this limitation is the incorporation of antibacterial additives, which can enhance and extend the sealer's antimicrobial action. Recent approaches suggest different antimicrobial mechanisms, such as the sustained release of antibacterial ions to target microbes within the dentinal tubules, or the use of contact-killing compounds to prevent colonization at the sealer-dentin interface (12).

The scientific literature has reported several compounds that impart antimicrobial properties when incorporated into endodontic sealers. Several narrative review articles have discussed the use of antimicrobial endodontic sealers in dentistry (9–12). However, critical appraisal of their efficiency and clinical applicability via systematically designed review to comprehensively illustrate their efficiency and clinical applicability is needed. Therefore, this review aimed to comprehensively evaluate antibacterial additives in resin-based antimicrobial endodontic sealers and examine their impact on antibacterial efficacy, physicochemical properties, and cytotoxicity. This review focused on adding antimicrobial compounds only in resin-based sealers, as they are the most used sealers in dentistry. In addition, this review aimed to develop several recommendations for future investigations on antimicrobial endodontic sealers, which can guide the better optimization and characterization of the new generations of antibacterial endodontic sealers.

## 2 Methodology

### 2.1 Design

This scoping review was conducted in accordance with the Preferred Reporting Items for Systematic Reviews and Meta-Analyses extension for Scoping Reviews (PRISMA-ScR) guidelines. The protocol for this review was registered retrospectively at PROSPERO (Registration ID: CRD420261333143) after completion of the review. Scanning of the available literature concerning antibacterial additives in resin-based antimicrobial endodontic sealers was achieved utilizing the 5-stage framework was achieved. Arksey and O'Malley's (13) and Levac, Colquhoun, and O'Brien (14) guidelines were used for articles screening, reviewing, and data examination. The 5-stage framework that was used involved: research question formulation, literature identification, selecting studies, study selection, and data analysis & outcomes reporting.

### 2.2 Research question

The review's questions were: “What are the antimicrobial additives in resin-based endodontic sealers?”, “What are the effects of the antimicrobial additives on physicochemical properties (e.g., solubility, setting time, bond strength) of resin-based sealers?” and “Do antimicrobial-modified resin-based sealers exhibit acceptable cytotoxicity profiles?”. These questions formulate the PICO criteria of this review as:

- Population (P): Resin-based endodontic sealers used in root canal obturation
- Intervention (I): Incorporation of antimicrobial additives (e.g., nanoparticles, quaternary ammonium compounds, antibiotics, natural extracts) into resin-based sealers
- Comparison (C): Unmodified commercial resin-based endodontic sealers
- Outcomes (O): Antimicrobial efficacy, physicochemical properties (e.g., setting time, solubility, bond strength, flow), and cytotoxicity profiles
- Type of Study (T): *in vitro* laboratory studies, *in vivo* animal studies, and *ex vivo* experimental investigations evaluating antimicrobial additives in resin-based endodontic sealers

### 2.3 Identifying the pertinent studies (search strategy)

Two authors (F.H. and M.G.) conducted an independent and comprehensive search of five online databases: Cochrane Library, PubMed, MEDLINE, Scopus, and Web of Science. They utilized a combination of MeSH terms and keywords in the search, as illustrated in Table 1. The search was done on November 23, 2025. Additionally, a manual search was conducted in primary journals of dental science and endodontics to address papers that were not available in the digital databases. Many articles designated as either early view, in press, or accepted were found through searches of the online portals of these journals. The search technique was applied in accordance with PRISMA standards (15). In addition to the electronic database searches, grey literature was searched to minimize publication bias and ensure comprehensive identification of all relevant evidence. Furthermore, the reference lists of all included studies were manually screened to identify any additional relevant studies not captured through the electronic database searches. The eligibility criteria were defined using the PICOS framework (Supplementary Table S1), specifying population, intervention, comparator, outcomes, and study design. Prior to the submission of this manuscript, an updated search was conducted on March 12, 2026, to identify any newly published relevant studies. The updated search showed no additional studies that met the predefined inclusion criteria.

**Table 1:** Keywords in different databases.

Database	Search strategy/keywords	Notes
PubMed	(“resin-based endodontic sealer” OR “epoxy resin sealer” OR “ antimicrobial endodontic material”) AND (“antibacterial additive” OR “antimicrobial efficacy” OR “biofilm inhibition” OR “root canal sealing” OR “physicochemical properties” OR “cytotoxicity assessment”).	Combination of MeSH and free-text terms; remineralization-related terms were excluded.
MEDLINE (via Ovid)	(resin-based endodontic sealer OR epoxy resin sealer OR antimicrobial endodontic material) AND (antibacterial additive OR antimicrobial efficacy OR biofilm inhibition OR root canal sealing OR physicochemical properties OR cytotoxicity assessment).	Search adapted to Ovid syntax; focus limited to antibacterial additives.
Scopus	TITLE-ABS-KEY((“resin-based endodontic sealer” OR “epoxy resin sealer” OR “ antimicrobial endodontic material”) AND (“antibacterial additive” OR “antimicrobial efficacy” OR “biofilm inhibition” OR “root canal sealing” OR “physicochemical properties” OR “cytotoxicity assessment”)).	Boolean operators adapted for Scopus search field syntax.
Web of Science	TS = ((“resin-based endodontic sealer” OR “epoxy resin sealer” OR “ antimicrobial endodontic material”) AND (“antibacterial additive” OR “antimicrobial efficacy” OR “biofilm inhibition” OR “root canal sealing” OR “physicochemical properties” OR “cytotoxicity assessment”)).	Topic search (TS) includes title, abstract, and author keywords.
Cochrane Library	(“resin-based endodontic sealer” OR “epoxy resin sealer” OR “ antimicrobial endodontic material”) AND (“antibacterial additive” OR “antimicrobial efficacy” OR “biofilm inhibition” OR “root canal sealing” OR “physicochemical properties” OR “cytotoxicity assessment”).	Search restricted to reviews and trials relevant to antibacterial additives in endodontic sealers.

### 2.4 Selecting eligible studies

The articles included in this review focused on the antibacterial additives in resin-based endodontic sealers and their associated cytotoxic and physicochemical effects. To exclude papers unrelated to the topic, the summaries and titles of the identified articles were reviewed. We obtained the full articles of the remaining publications for future examinations. Additional relevant studies were identified by searching for the references for the selected articles. The inclusion criteria were peer-reviewed studies, studies that evaluated the antibacterial properties of resin-based endodontic sealers, used an appropriate control group, and were published in English. The exclusion criteria were studies that used non–resin-based sealers, lacked a control group, were not in English, or were conference papers, abstracts, or grey literature. Studies that did not test the antibacterial properties of the sealers using valid antibacterial assays were also excluded. The records retrieved from the searched databases were uploaded to Rayyan (https://www.rayyan.ai), a web-based platform utilized for study screening and data management. The screening process was performed by two independent reviewers (F.H. and M.G.). The procedure included an initial screening by summary and title, then a complete text screening. Any conflicts among the reviewers were adjusted by a third reviewer (W.M.). A conflict was defined as any disagreement between the two reviewers regarding the decision to include or exclude a study from the analysis. Inter-rater reliability was assessed using Cohen's Kappa statistics, with a value greater than 0.8 achieved, indicating almost perfect agreement between the reviewers.

### 2.5 Data charting

A datasheet was developed to compile all collected data and document the findings for each parameter and variable. PRISMA flowchart was used to show the sequence of data collection and screening.

### 2.6 Data extraction & reporting results

Data was extracted by two independent reviewers (F.H. and M.G.) using a standardized data extraction form developed in Microsoft Excel (Microsoft Corporation, Redmond, WA, USA). The following data were extracted: author name, year of publication, antibacterial additives, methods for studying antibacterial activity, physicochemical properties, and main findings of the study. Any discrepancies between the two reviewers were resolved through discussion and consensus, with the involvement of a third reviewer (W.M) when necessary.

### 2.7 Risk of bias assessment

The risk of bias for all 33 included *in vitro* studies was assessed using the Modified Jadad Scale, which comprises 18 items covering methodological quality domains such as randomization, blinding, sample size justification, controls, replication, statistical analysis, outcome clarity, selective reporting, conflict of interest and funding disclosure, cytotoxicity assessment, material characterization, method standardization, and handling of incomplete data, with a maximum possible score of 18. Studies scoring 14 or above were classified as low risk, scores between 9 and 13 as moderate risk, and scores below 9 as high risk. The Modified Jadad Scale was selected for risk of bias assessment due to its suitability for evaluating *in vitro* and laboratory-based experimental studies, which constituted all the included studies in this review. Unlike tools designed primarily for clinical trials, this Scale provides a practical and validated framework for assessing methodological quality parameters relevant to laboratory investigations, making it an appropriate choice for the study designs included in this scoping review.

## 3 Results

### 3.1 Included articles

**Figure 1:**
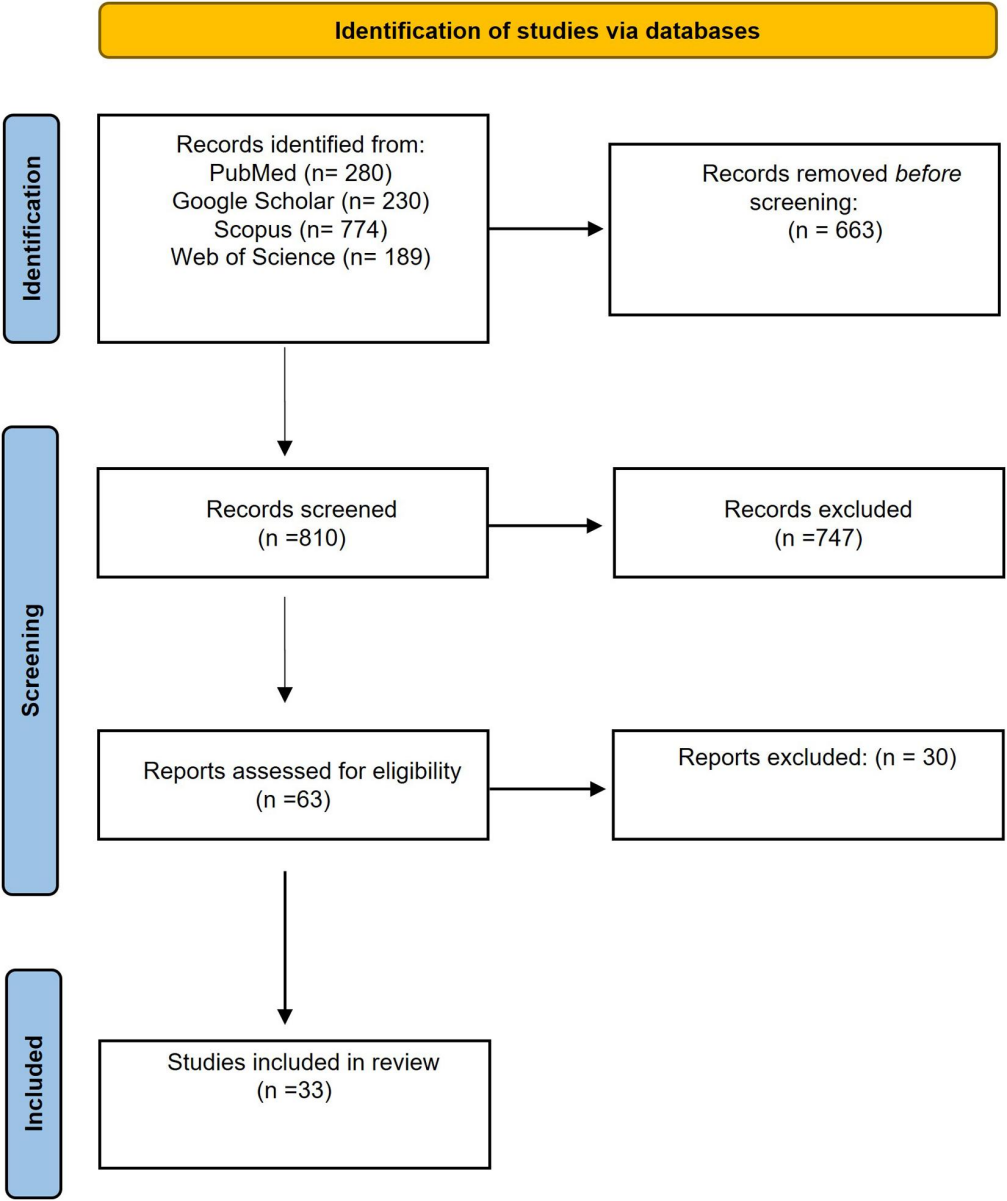
PRISMA chart for the included studies.

After searching the specified databases mentioned earlier in the Methodology section, 1,473 research papers were found. We examined 810 papers after excluding 663 duplicates. The papers were then examined at the title and abstract level, and 747 papers were excluded, leaving 63 papers. These remaining papers were fully downloaded, and 30 more were subsequently excluded. Therefore, the number of papers included in this review is 33, as clearly shown in (Figure 1).

### 3.2 Main characteristics of the included studies

Table 2 summarizes the endodontic sealers used across all 33 included studies. AH Plus was the most frequently used base sealer (*n* = 19), followed by MTA Fillapex (*n* = 3), BioRoot RCS (*n* = 3), and AH 26 (*n* = 2), while eight studies employed fully experimental sealer formulations.

**Table 2:** Endodontic sealer used across All included studies.

Study	Category	Antimicrobial additive	Endodontic sealer used
Zebari et al., 2023 (23)	Herbal Extracts	Thymus Kotschyanus Boiss	BioRoot™ RCS (modified); AH Plus (control)
Devi et al., 2019 (25)	Herbal Extracts	Salvadora persica, Emblica officinalis, Myristica fragrans	Endomethasone, AH Plus, and Apexit Plus
Saha et al., 2019 (24)	Herbal Extracts	Glycyrrhiza glabra, Mimusops elengi, Tinospora cordifolia	Zinc oxide eugenol-, epoxy resin-, and calcium hydroxide-based sealers
Wahyuni et al., 2023 (113)	Herbal Extracts	Propolis nanoparticles (5%)	Epoxy resin-based and bioceramic sealers
Qi et al., 2023 (101)	Herbal Extracts	Piper betle extract	AH Plus and BioRoot RCS
Vilela Teixeira et al., 2019 (76)	Nanoparticles/Nanomaterials	Silver Vanadate (AgVO₃)	AH Plus, Sealer 26, and Endomethasone N
Ibrahim and Al-Huwaizi, 2023 (114)	Nanoparticles/Nanomaterials	Nano-sized amorphous calcium phosphate (NACP)	AH Plus
Hassan et al., 2024 (93)	Nanoparticles/Nanomaterials	Silver-gold nanoparticles	AH Plus and Nano Care Gold
Roshdy et al., 2021 (115)	Nanoparticles/Nanomaterials	Silver nanoparticles (AgNPs)	AH Plus and Ceraseal
Farahat et al., 2022 (68)	Nanoparticles/Nanomaterials	Silver nanoparticles (AgNPs)	AD Seal, MTA Fillapex, and GuttaFlow
Torres-Betancourt et al., 2022 (27)	Nanoparticles/Nanomaterials	Bismuth lipophilic nanoparticles (BisBAL NPs)	AH Plus
Meng et al., 2020 (31)	Nanoparticles/Nanomaterials	Nano-magnesium hydroxide (NMH)	AH Plus
Alharamlah et al., 2025 (36)	Quaternary Ammonium Compounds	DMAHDM (5%)	AH Plus
Liu et al., 2019 (37)	Quaternary Ammonium Compounds	DMADDM	EndoREZ
Baras et al., 2019 (84)	Quaternary Ammonium Compounds	DMAHDM, NAg, and NACP	Experimental methacrylate resin sealer
Liu et al., 2024 (82)	Quaternary Ammonium Compounds	Quaternary ammonium polymethacrylate bioactive glasses (BGs-HAEM)	Experimental resin sealer
Baras et al., 2019 (116)	Quaternary Ammonium Compounds	BTH + 73glass + DMAHDM	Experimental sealer; AH Plus (control)
Seung et al., 2018 (117)	Quaternary Ammonium Compounds	DMAHDM and NAg	AH Plus
Arias-Moliz et al., 2015 (38)	Quaternary Ammonium Compounds	Benzalkonium chloride	AH Plus
Monteiro et al., 2019 (33)	Other Additives	ATAB-loaded halloysite nanotubes (ATAB/HNT)	Experimental resin sealer
Weckwerth et al., 2015 (118)	Other Additives	Antifungal drugs	AH Plus, MTA Fillapex, Sealapex, Sealer 26, and EndoFill
Ghaffari et al., 2024 (119)	Other Additives	Nanosilver, chitosan, and propolis	Nano zinc oxide eugenol (nZOE) sealer, AH Plus
Khoshbin et al., 2025 (61)	Other Additives	Chitosan nanoparticles (CsNPs) and arginine	AH Plus
Kangarlou et al., 2016 (83)	Other Additives	Amoxicillin, triple antibiotic paste, and nanosilver	AH26 and AH Plus
Collares et al., 2018 (77)	Other Additives	Chlorhexidine and *α*-tricalcium phosphate (α-TCP)	Experimental methacrylate-based sealer
Loyola-Rodríguez et al., 2019 (63)	Other Additives	Chitosan (CsNPs) and silver nanoparticles (AgNPs)	AH Plus, Endosequence, MTA Fillapex, Sealapex, and Tubliseal
Ratih et al., 2023 (81)	Other Additives	Chitosan nanoparticles	AH 26
Mokabberi et al., 2024 (120)	Other Additives	Triple antibiotic paste (TAP)	AH Plus
Habib et al., 2021 (86)	Other Additives	Green tea extract–chitosan microcapsules	Experimental epoxy resin-based sealer
Kapralos et al., 2022 (50)	Other Additives	Chlorhexidine digluconate	AH Plus, BioRoot RCS, and Pulp Canal Sealer (PCS)
Rossato et al., 2017 (121)	Other Additives	Dibutyltin (ET) or calcium (EC) methacrylate	Experimental resin sealer; RealSeal (base)
Carvalho et al., 2021 (58)	Other Additives	CHX-HMP nanoparticles	AH Plus, MTA Fillapex, and Pulp Canal Sealer
Del Carpio-Perochena et al., 2022 (122)	Other Additives	Chitosan-hydroxyapatite nanocomplexes	AH Plus

The biologically active compounds incorporated into resin-based endodontic sealers have been classified into four main categories: Category 1 includes: herbal extracts (Table 3), Category 2 includes: inorganic nanoparticles and nanotubes (Table 4), Category 3 includes: quaternary ammonium compounds with and without metallic compounds (Table 5), and Category 4 includes: other agents including chemical agents, antibiotics, or a combination between the different approaches (Table 6).

**Table 3:** Herbal extracts of antimicrobial additives characteristics of the included studies.

Study ID	Antimicrobial additive (s)	Methods of studying the antibacterial activity	Physicochemical properties	Main findings
Zebari et al., 2023 (23)	Thymus Kotschyanus Boiss (herbal extract)	Agar diffusion test (ADT)	Not evaluated	The AH Plus sealer and experimental material (1–1) % shown a superior antibacterial effect compared to the BioRootTM sealer. The oil of Thymus Kotschyanus Boiss may be included as an antibacterial agent in root canal sealers.
Devi et al., 2019 (25)	There were three different herbal extracts: Salvadora persica (Miswak), Emblica officinalis (Amla), as well as Myristica fragrans (Nutmeg).	Agar diffusion test	Not evaluated	Zinc oxide eugenol, epoxy resin, or calcium hydroxide dental canal sealers have their cytotoxicity and antibacterial activity mitigated by the addition of extracts from [Emblica officinalis, Amla], Myristica fragrans, Nutmeg, Salvadora persica, and miswak.
Saha et al., 2019 (24)	Extracts from the herbs licorice (Glycyrrhiza glabra), bazul (Mimusops elengi), and guduchi (Tinospora cordifolia).	Agar Diffusion Test	Not evaluated	The sealer that contained zinc oxide eugenol demonstrated the most significant statistically significant zones of bacterial growth suppression when it was combined with Glycyrrhiza glabra (licorice), Tinospora cordifolia (Guduchi), & Mimusops elengi (Bakul), in that order.
Wahyuni et al., 2023 (113)	5% of propolis nanoparticle	CFU	Not evaluated	Commercial sealers made from bioceramic and epoxy resin types have enhanced their antibacterial qualities when five percent propolis nanoparticles were added.
Qi et al., 2023 (101)	Piper betle (PB)	Modified direct contact test (MDCT)	Cytotoxicity	The cytotoxicity of PB and AH plus (PBAH) was significantly lower than that of AH. Both AH as well as PBAH exhibited antibacterial properties against E. faecalis.

**Table 4:** The characteristics of the included studies utilizing inorganic nanoparticles and metallic compounds.

Study ID	Antibacterial additive (s)	Methods of studying the antibacterial activity	Physicochemical properties	Main findings
Vilela Teixeira et al., 2019 (76)	Silver Vanadate (AgVO_3_), at 2.5%, 5%, and 10%	Colony-forming unit (CFU) count (spectrophotometry)	Setting Time, Chemical composition,	The Silver Vanadate was integrated into AH Plus, Sealer 26, and Endomethasone N at concentrations of 0%, 2.5%, 5%, & 10 percent, as well as all groups demonstrated inhibition of E. faecalis. Silver Vanadate of AH Plus exhibited a reduced setting time compared to the control group. AgVO3 elevated the atomic percentages of Ag & V, altering the atomic percentages of the sealer components in addition to the setting time.
Ibrahim and Al-Huwaizi, 2023 (114)	Nano-sized amorphous calcium phosphate (NACP)	Agar diffusion test	Flowability, PH Calcium and phosphate release	AH Plus + NACP exhibited superior antibacterial efficacy compared to AH Plus. Both materials demonstrated no significant change relative to the negative control, indicating reduced cytotoxicity. The sealer, which contained NACP, was able to neutralize acidity, raise pH levels, and release calcium and phosphate ions all while keeping its flowability.
Hassan et al., 2024 (93)	Silver gold nanoparticles	ADT	Cytotoxicity	After 24 h, no significant distinction was observed in the diameter of the inhibitory zones across the groups. Measurements taken over forty-eight hours indicated that zones created with Nanogold had considerably greater inhibitory zone diameters compared to those developed with AH Plus when examined independently. After seventy-two hours, the inhibition zone values of Nano gold were substantially greater than those of the AH Plus and Nano gold combo. Minimal cytotoxicity.
Roshdy et al., 2021 (115)	Silver nanoparticles	DCT	Not evaluated	The antibacterial activity of both AH Plus and Ceraseal sealer can be enhanced by the addition of silver nanoparticles (AgNPs). At various time intervals, AH Plus demonstrated more potent antibacterial activity against Enterococcus faecalis in comparison to Ceraseal sealer, which was not influenced by the addition of AgNPs.
Farahat et al., 2022 (68)	Silver nanoparticles	DCT	Not evaluated	The antibacterial impact of all sealers was enhanced by adding silver nanoparticles.
Torres-Betancourt et al., 2022 (27)	Bismuth lipophilic nanoparticles (BisBAL NPs)	Disc diffusion assays.	Cytotoxicity	The antibacterial activity of AH Plus alone is 4.9 times lower than that of AH Plus supplemented with BisBAL NP. A novel nanomaterial, BisBAL NP-supplemented AH Plus, can be used to protect endodontic patients from re-infection without causing cytotoxicity.
Meng et al., 2020 (31)	Nano-magnesium hydroxide (NMH)	MDCT	Not evaluated	The antimicrobial activity of NMH powder against S. mutans was exceptional. AH Plus, when combined with 5% or 7% NMH, was more effective against S. mutans than AH Plus alone (*P* < 0.05). The solidified AH Plus maintained its antibacterial properties on the seventh day due to the presence of NMH.

**Table 5:** Antimicrobial monomers and quaternary ammonium additives’ characteristics of the included studies with and without inorganic or metallic compounds.

Study ID	Antibacterial additive (s)	Methods of studying the antibacterial activity	Physicochemical properties	Main findings
Seung et al., 2018 (117)	Quaternary ammonium compound, nanosilver (0.15% NAg) & dimethylaminohexadecyl methacrylate (2.5% DMAHDM).	Modified DCT, CFU count	Solubility, flow, Setting time, as well as dimensional change	The physical properties of the sealer were not affected by the addition of 2.5 percent DMAHDM as well as 0.15 percent NAg to AH Plus. In contrast to AH Plus, the modified AH Plus exhibited a substantially increased antibacterial efficacy against E. faecalis fourteen days after setup.
Baras et al., 2019 (84)	DMAHDM (0%, 2.5% and 5%,) and NAg (0.05%, 0.1% and 0.15%)	CFU count, polysaccharide production by the biofilms	Flow, film thickness, color, and sealing ability	In contrast to AH Plus and the control group, the biofilms treated with 5 percent DMAHDM in addition to 0.15 percent % NAg reduced their CFU by more than six orders of magnitude and produced less polysaccharide. DMAHDM as well as NAg did not adversely impact the film thickness as well as sealing characteristics.
Liu et al., 2024 (82)	Quaternary ammonium polymethacrylate (QAPM)-containing antimicrobial glasses (BGs) namely BGs-HAEM.	ADT, DCT, CLSM	Not evaluated	After fourteen and twenty-eight days of incubation, BGs-HAEMB showed the strongest antibacterial activity against E. faecalis, S. sanguis, & P. endodontalis, contrasted with Endofill, AH Plus, as well as iRoot SP. The antimicrobial efficacy of BGs-HAEMB was the highest among all experimental groups, regardless of the duration of treatment, including seven days and twenty-eight days. The BGsHAEMB-treated groups exhibited comparatively low cytotoxicity, with relative growth rates (RGRs) ranging from 88.6 percent to 102.9% after one, three, and seven days of exposure.
Baras et al., 2019 (116)	5% DMAHDM, 0.15 percent NAg, and NACP at 10 percent, 20 percent and 30 percent mass fractions	Colony-forming units (CFU) count	Flow, film thickness, Ca and P ion release, PH, and Dentin hardness	DMAHDM, NAg, and NACP, the three antimicrobial agents, neutralized acid, elevated pH, restored dentin minerals, strengthened root dentin, as well as decreased dentin-block-impregnated biofilm CFU by three logs.
Liu et al., 2019 (37)	DMADDM (0, 1.25%, 2.5%, in addition to 5%)	Assessment of colony-forming units by DCT	Cytotoxicity, and solubility	Sealers containing DMADDM (1.25 percent, 2.5 percent) & EndoREZ (0%) had no noticeable distinction in terms of cytotoxicity, apical sealing capacity, and solubility. In multispecies microecology, sealers containing DMADDM could lower the composition ratio of E. faecalis. The antibacterial properties of EndoREZ could be significantly enhanced by the incorporation of DMADDM into the sealer.
Arias-Moliz et al., 2015 (38)	Benzalkonium chloride (BC).	DCT and confocal laser scanning microscopy.	Setting time, flow, solubility and microhardness testing	Physical qualities stated in ANSI/ADA standards were unaffected by the addition of BC to AH Plus at concentrations of two percent or higher, which demonstrated antibacterial & antibiofilm actions.
Alharamlah et al., 2025 (36)	DMAHDM	Colony-forming units (CFUs) and scanning electron microscopy (SEM).	Film thickness, solubility, contact angle, and flow.	Incorporating 5wt. % DMADDM slightly altered the sealers’ physical properties but kept them within clinically acceptable limits. Completely eradicated E.faecalis biofilms, unlike the control sealers, which showed heavy bacterial growth.

**Table 6:** List of studies utilizing dual antimicrobial agents or chemical additives’ features.

Study ID	Antibacterial additive (s)	Methods of studying the antibacterial activity	Physicochemical properties	Main findings
Carvalho et al., 2021 (58)	Chlorhexidine-hexametaphosphate nanoparticles (CHX-HMP NPs)	DCT and CFU count	Cytotoxicity, setting time, flow, radiopacity, solubility and pH	AH Plus (AH), MTA Fillapex (MTA), as well as PCS all shown an enhancement in their antibacterial effect in the DCT. The sealers reduced flow and increased solubility after 24 h of immersion; however, they had no effect on the samples’ radiopacity. The setting time for AH rose, while MTA failed to reach setting under any of the studied conditions. The pH value of all samples decreased as the immersion time went on. Endodontic sealers can have their antimicrobial effectiveness enhanced by adding NPs, and this is all without sacrificing any of the other biological or physicochemical qualities of the sealer.
Kapralos et al., 2022 (50)	Chlorhexidine (CHX)	A DCT, CFU count	PH, water uptake, solubility, porosity, sorption, surface characteristics	Chlorhexidine enhanced the antibacterial characteristics Pulp Canal Sealer (PCS) exhibited the most significant alteration in physical attributes due to CHX, while AH Plus shown an increase in solubility. Chlorhexidine did not influence the physical features of BioRoot RCS, with the exception of a reduction in solubility.
Habib et al., 2021 (86)	Green tea-chitosan microcapsules	DCT, optical density	Setting Time, Film Thickness, Solubility, Microleakage Evaluation, PH	The GTE-CSNPs microcapsule demonstrated considerable enhancement in the antibacterial efficacy as well as sealing capacity of epoxy resin-based endodontic sealers. The setting time, film thickness, along with microleakage were reduced, but solubility was elevated.
Mokabberi et al., 2024 (120)	Triple antibiotics paste combination (metronidazole, minocycline & ciprofloxacin)	CFU Count	Not evaluated	No substantial variations were seen in either the 1-day or 3-day sets of the fresh antibiotic-AH Plus sealer combination (*P* = 0.525). Nevertheless, compared to the other sets, the average CFU/mL in the sealer with the 7-day set was significantly lower (*P* under 0.001). The one percent triple antibiotic was the most effective concentration for inhibiting bacterial growth. All concentrations (1%, 5%, 10%, in addition to 25%) effectively inhibited bacterial growth, confirming that the antibacterial efficacy increases with higher antibiotic concentrations. There was not a significant variance in the average growth decrease across various concentrations (one, five, ten, and twenty-five percent).
Ratih et al., 2023 (81)	The concentrations of chitosan nanoparticles are zero, ten, twenty as well as thirty percent.	Agar diffusion test	Cytotoxicity	Greater antibacterial activity and decreased cytotoxicity were seen when epoxy resin-based (ERB) sealer containing chitosan nanoparticles was added at doses of 10, 20, and 30%, as compared to a control group that did not receive any chitosan.
Loyola-Rodríguez et al., 2019 (63)	Chitosan (CsNPs), silver nanoparticles (AgNPs), calcium hydroxide with propylene glycol, (Ca (OH)_2_ + propygly); chlorhexidine (Chx) (at 0.3%); and CsNPs containing Chx (CsNPs-Chx).	Monolayers on agar plates and tests against E. faecalis on the surface of collagen membranes were employed to determine the bactericidal activity.	Not evaluated	The antibacterial activity increment (AAI) of the CsNPs-Chx combination was the highest at 55%, followed by Chx at 35.5% and CsNPs at 11.1%. Conventional endodontic treatments could be utilized to manage E. faecalis bacteria through the use of Tubliseal and AH plus sealers in conjunction with nanoparticles, particularly CsNPs-Chx.
Collares et al., 2018 (77)	Chlorhexidine and a-tricalcium phosphate (a-TCP)	Agar diffusion test	Flow, film thickness, radiopacity, degree of conversion (DC), degradation in water, and pH	After 28 days of water immersion, the groups that contained 5% CHX had the lowest pH values and the maximum weight loss (WL). When evaluated against Enterococcus faecalis, all chlorhexidine-containing solutions proved to be effective antibacterial agents. The physicochemical parameters and antibacterial properties of an experimental root canal sealer based on methacrylate were affected by the addition of CHX and a-TCP.
Kangarlou et al., 2016 (83)	Amoxicillin, triple antibiotic pastes and Nano silver	Agar diffusion test	Not evaluated	The bactericidal effects of the sealers were not enhanced by nano silver. The bactericidal efficacy of sealers combined with amoxicillin was strongest in fresh conditions. The antibacterial capabilities of AH Plus and AH26 sealers were greatly enhanced by amoxicillin and triple antibiotic paste.
Khoshbin et al., 2025 (61)	Chitosan nanoparticles (CsNPs) and arginine (Arg)	Agar diffusion test.	Size, morphology, chemical structure, and cytotoxicity	Increased antimicrobial and antibiofilm activity against Enterobacter faecalis and Candida albicans is a result of the addition of CsNPs as well as Arg to AH Plus sealer.
Ghaffari et al., 2024 (119)	Chitosan, Propolis, and Nano silver	Agar diffusion as well as broth microdilution tests.	Not evaluated	Despite the addition of nano-silver, chitosan, and propolis to nZOE and AH Plus, the diameter of the growth inhibition zone in the agar diffusion test did not change by any means. When propolis and eugenol were employed on their own, the minimum inhibitory concentration (MIC) and minimum bactericidal concentration (MBC) were found to be minimum. The utilization of chitosan by itself resulted in the highest MIC as well as MBC concentrations being reported. In comparison to micro-ZOE, nZOE had a smaller MBC (*P* = 0.000). The MIC and MBC values were the lowest in all groups that contained nZOE. nZOE's antibacterial action against E. faecalis *in vitro* can be enhanced by adding propolis to it.
Weckwerth et al., 2015 (118)	Antifungal drugs (ketoconazole and fluconazole)	Agar diffusion test	Setting time and flowability	Antifungal drugs improved the antimicrobial action of most cements tested without changing their physical properties. When added to endodontic sealers (Sealer 26, AH Plus, Endofill, Fillapex, as well as Sealapex), these drugs had no effect on the setting time or flowability of the sealers.
Monteiro et al., 2019 (33)	ATAB/HNT	Direct contact inhibition assay & Planktonic bacteria inhibition assay	Radiopacity, flow, film thickness and Cytotoxicity	Experimental resin sealers that included ATAB/HNT had antibacterial effects against biofilm and planktonic E. faecalis, but these effects had no effect on the chemo-mechanical characteristics or pulp cell viability.
Del Carpio-Perochena et al., 2022 (122)	Chitosan-hydroxyapatite (CS-HA)	CFU Count	Flowability, solubility, Morphology, Fracture resistance, Hardness and modulus of elasticity	Following four weeks of immersion, the TCS/CS-HA tricalcium silicate sealer exhibited ideal flow characteristics without influencing solubility. The nanohardness and elastic modulus were both significantly improved by TCS/CS-HA. From the initial bacterial load, CS-HA reduced bacteria by 2.04 logarithms. The use of TCS or TCS/CS-HA to fill root canals that had been conditioned with CS-HA resulted in an even greater decrease of germs. Root dentin was more resistant to fractures and had fewer residual germs after using CS-HA. The combination of TCS with CS-HA enhanced TCS's physical and nanomechanical characteristics.
Rossato et al., 2017 (121)	Methacrylate metal salts incorporated into the experimental sealers	MDCT, and cell viability.	Cytotoxicity	The experimental sealers’ antibacterial efficacy was enhanced by the addition of calcium and dibutyltin methacrylate. It appeared that a good treatment option that provided antibacterial activity with minimal cytotoxicity and sufficient physical qualities was the integration of calcium methacrylate at concentrations of 0.5 percent, one percent, and two percent.

ADT, agar diffusion test; BGs-HAEMB, antimicrobial glasses with quaternary ammonium polymethacrylate; Ca(OH)_2_, calcium hydroxide; Ca and P, calcium and phosphate; CFU, colony forming unit; CHX, chlorhexidine; CHX-HMP NPs, chlorhexidine-hexametaphosphate nanoparticles; CLSM, confocal laser scanning microscopy; CS-HA, chitosan-hydroxyapatite; CsNPs, chitosan nanoparticles; DMAHDM, dimethylaminohexadecyl methacrylate; DMADDM, dimethylaminododecyl dimethacrylate; DCT, direct contact test; EC, calcium methacrylate; ERB, epoxy resin-based; ET, dibutyltin methacrylate; GTE-CSNPs, green tea extract-chitosan nanoparticles; HNT, halloysite nanotubes; ATAB, alkyl trimethyl ammonium bromide; MDCT, modified direct contact test; MIC, minimum inhibitory concentration; MBC, minimum bactericidal concentration; NACP, nanoparticles of amorphous calcium phosphate; NAg, nano-silver; NMH, nano-magnesium hydroxide; NPs, nanoparticles; PB, piper betle; PBAH, piper betle + AH plus; PCS, pulp canal sealer; QAPM, quaternary ammonium polymethacrylate; RGR, relative growth rate; TCS, tricalcium silicate; ZOE, zinc oxide eugenol.

**Figure 2:**
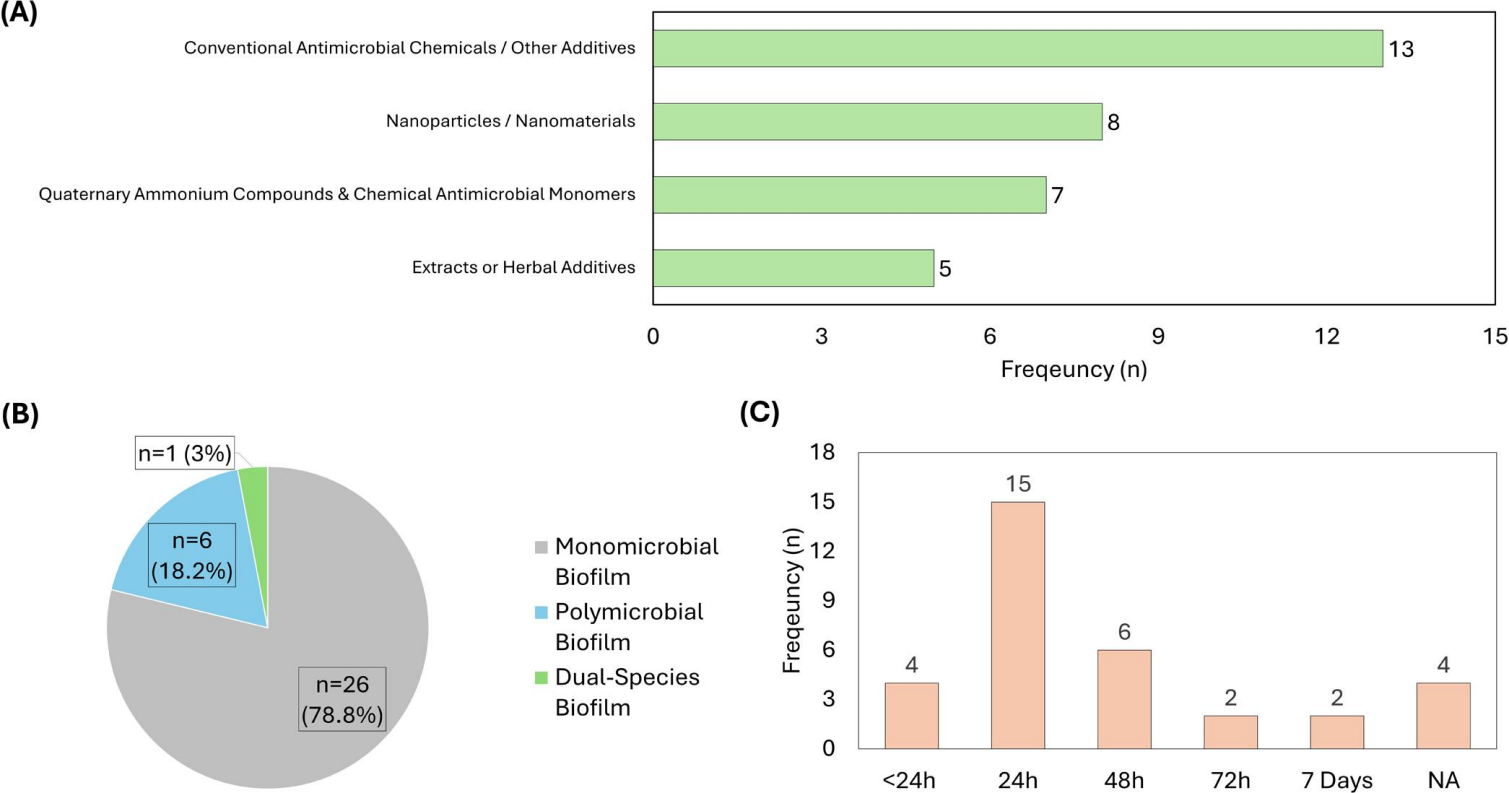
Main characteristics of the developed sealers and the developed biofilms. **(A)** Classification of antimicrobial additives evaluated in resin-based endodontic sealers across studies included in this review (*N* = 33). **(B)** The type of biofilms used in the study in relation to the species number. **(C)** Duration of the biofilm development.

**Figure 3:**
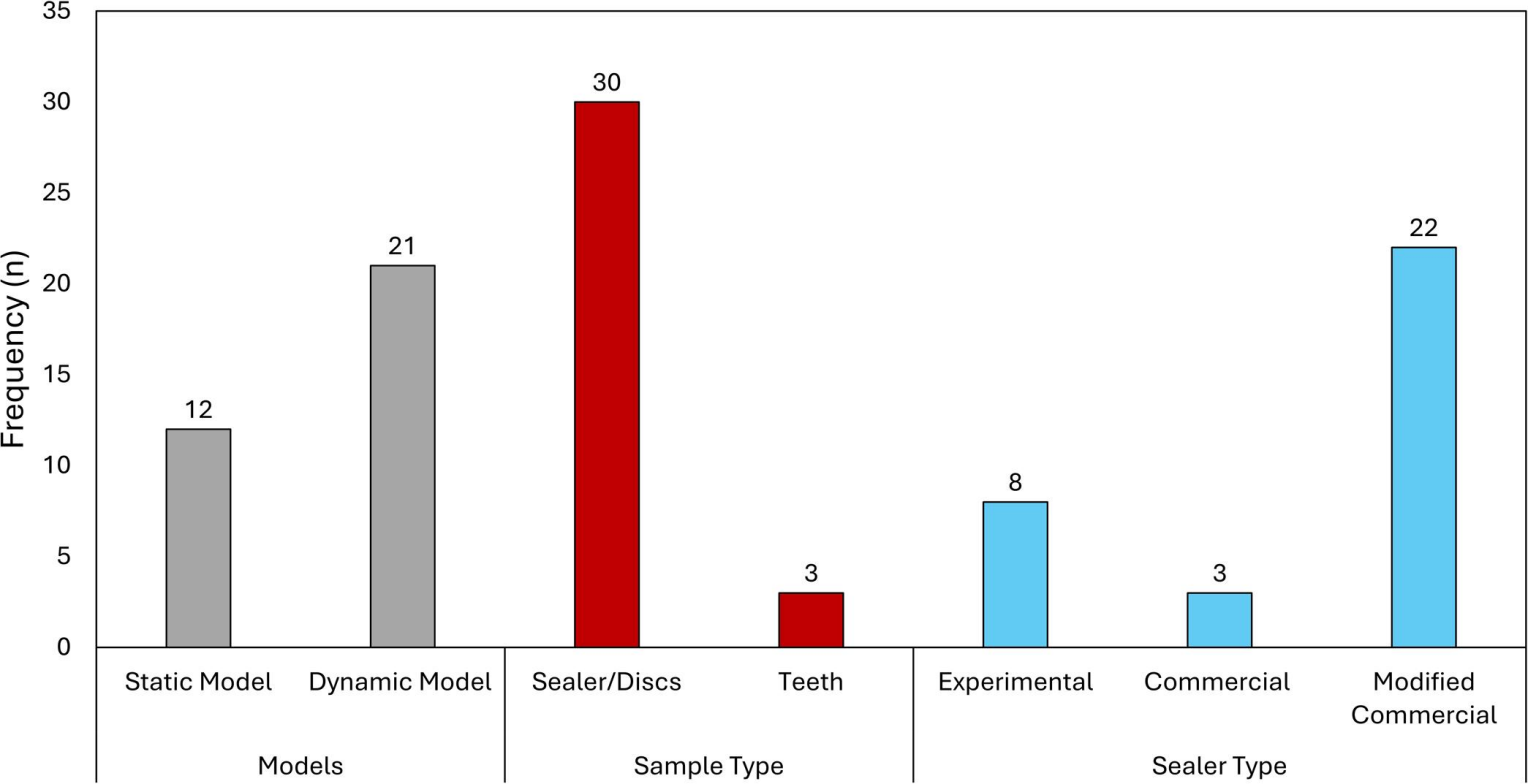
Distribution of experimental models, sample types, and sealer categories used among included studies (*N* = 33).

**Figure 4:**
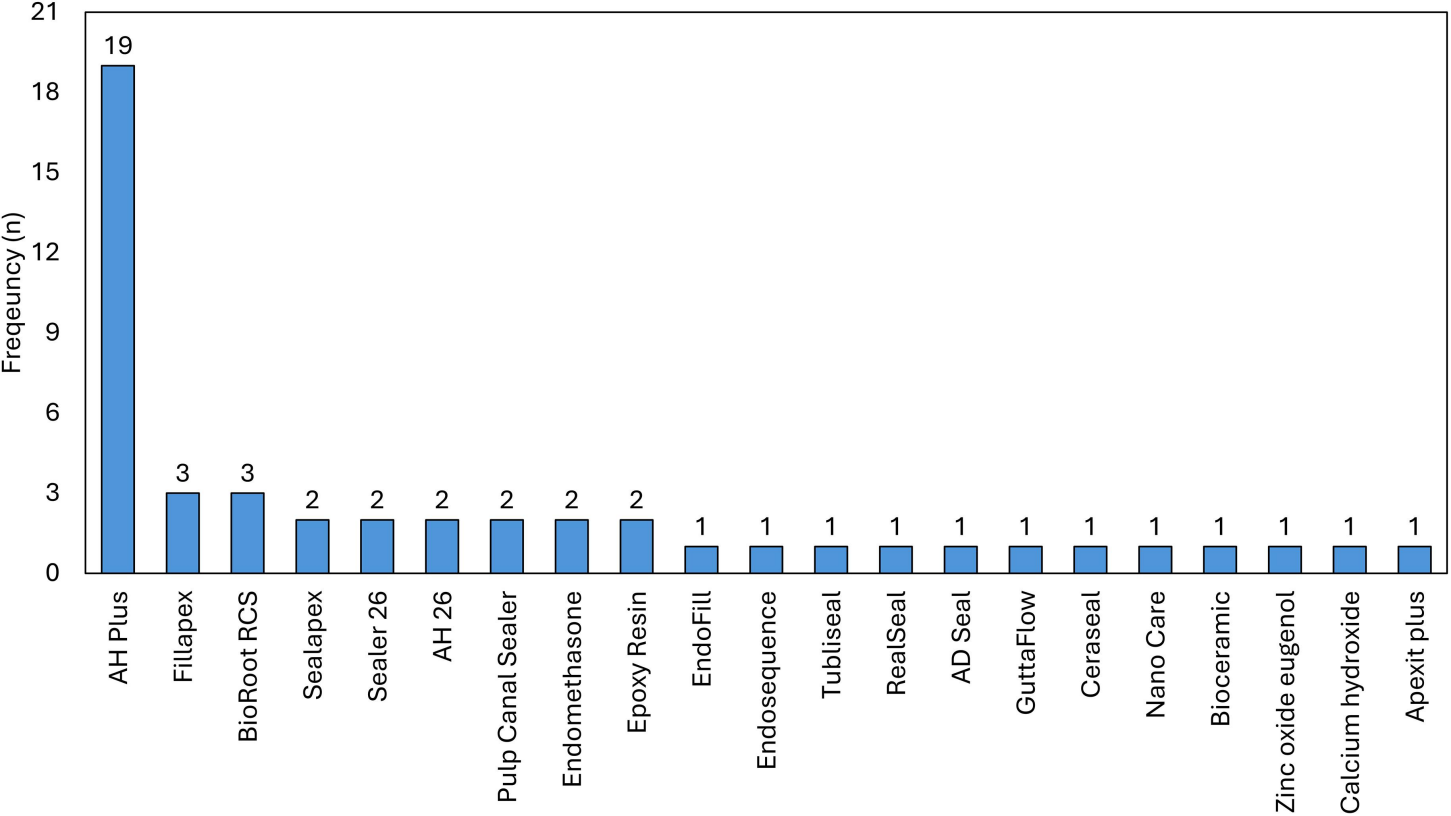
Frequency of commercial endodontic sealers tested with multiple experimental groups among included studies (*N* = 33).

Figure 2 illustrates the main characteristics of the antimicrobial compounds used in endodontic sealers and the developed biofilms. All studies took place *in vitro*. Most of the included studies have utilized chemical agents (*n* = 13) to add antimicrobial properties to endodontic sealers, followed by nanoparticles or nanomaterials (*n* = 8) (Figure 2A). Of the biofilm models, most (*n* = 26) used a monomicrobial system, especially *Enterococcus faecalis* (Figure 2B). Most studies used biofilm models grown for 24 h (15 studies) (Figure 2C). Figure 3 displays that dynamic biofilm models was more commonly used than static models (21 vs. 12). Most studies (*n* = 30) used sealers discs for the biofilm assays, while three studies used extracted teeth. Regarding the sealer used, modified commercial sealers were most common (*n* = 22), while there were 8 experimental sealers and 3 commercial sealers (Figure 3). Finally, the most frequently used commercial control sealer was AH Plus (*n* = 19) (Figure 4).

### 3.3 Herbal extracts as antimicrobial additives

The herbal extracts studies showed enhancement of the antibacterial activity of the endodontic sealers (Table 3). Antibacterial activity was improved in the herbal extracts of *Thymus Kotschyanus*, *Salvadora Persica*, *Emblica Officinalis*, *Myristica Fragrans*, *Glycyrrhiza Glabra*, *Tinospora Cordifolia*, *Mimusops Elengi*, Propolis Nanoparticles, and Piper Betle, with some combinations also showing a reduction in cytotoxicity. Hence, it may be hypothesized that the pharmacological antimicrobial properties of the different herbal extracts could be incorporated into endodontic sealers with added antibacterial activity without evaluating their physicochemical properties.

### 3.4 Nanoparticles and nanomaterials as antimicrobial additives

The main findings for incorporating nanoparticles and nanomaterials (e.g., nanotubes) in endodontic sealers are shown in Table 4. Endodontic biofilms were inhibited when silver vanadate, silver/gold, nano-magnesium hydroxide, biophilic, bismuth nanoparticles, chlorhexidine-hexametaphosphate nanoparticles, and nano-sized amorphous calcium phosphate (NACP) were used. Some nanoparticles, such as AgVO₃ and NACP, influenced physicochemical features, including setting time, pH, and ion release. Other additives were found to improve the flow properties and antimicrobial activity of the sealers.‏

### 3.5 Chemical and quaternary ammonium antimicrobial additive

Sealers mixed with quaternary ammonium compounds, such as dimethylaminododecyl methacrylate (DMADDM), dimethylaminohexadecyl methacrylate (DMAHDM), benzalkonium chloride, and certain antimicrobial glasses, have shown greater enhancement of their antibacterial activity, especially against *E. faecalis* and multispecies biofilms (Table 5). These additives usually did not provide acceptable physicochemical characteristics such as flow, setting time, and solubility. In other multi-component formulations, i.e., DMAHDM, Nag, and NACP, the combination increased the pH, improved dentin hardness, and reduced biofilm formation.

### 3.6 Other antimicrobial additives

There are numerous additives (Table 6) that exhibit varying degrees of antibacterial effects against endodontic pathogens and impart more features to the endodontic sealers. These additives include chlorhexidine, chitosan nanomaterials, propolis, some antifungal drugs, antibiotic mixtures, a-TCP, methacrylate salts, chitosan-hydroxyapatite, and chlorhexidine. Some additives reduced the flow or solubility of the sealers, or simply maintained their physicochemical properties, while others affected solubility to some extent or flow in most systems.

### 3.7 Risk of bias

Table 7 demonstrates the risk of bias assessment for the included studies. Most of the included studies (27 out of 33) were rated as moderate risk of bias, with total scores ranging from 9 to 12, indicating generally acceptable but imperfect methodological quality. Six studies were classified as high risk of bias, with scores of 7 or 8, showing notable methodological limitations. No study achieved a low-risk classification. Across all studies, items related to randomization, blinding, and sample size justification were consistently underreported or absent, representing the most prevalent sources of bias. In contrast, most studies adequately reported control groups, replication, statistical analyses, and outcome measures, suggesting that while experimental rigor was partially maintained, transparency and reporting standards remain areas for improvement in this body of literature.

**Table 7:** Modified jadad scale for *in vitro* studies.

Study ID	Items																		Total score	Overall risk
	1	2	3	4	5	6	7	8	9	10	11	12	13	14	15	16	17	18		
Zebari et al., 2023 (23)	0	0	0	0	0	1	1	1	1	1	1	0	1	1	0	1	1	0	10	Moderate
Devi et al., 2019 (22)	1	0	0	0	0	1	1	0	1	1	1	1	0	0	0	1	1	1	10	Moderate
Saha et al., 2019 (24)	0	0	0	0	0	1	1	0	1	1	1	1	1	0	0	0	1	0	8	High
Wahyuni et al., 2023 (113)	1	0	0	0	0	1	1	0	1	1	1	1	0	0	0	1	1	0	9	Moderate
Qi et al., 2023 (101)	0	0	0	0	0	1	1	1	1	1	1	1	0	0	1	1	1	0	10	Moderate
Vilela Teixeira et al., 2019 (76)	0	0	0	0	0	1	1	0	1	1	1	1	0	1	0	1	1	0	9	Moderate
Ibrahim and Al-Huwaizi, 2023 (114)	0	0	0	0	0	1	1	1	1	1	1	1	1	1	1	1	1	0	12	Moderate
Hassan et al., 2024 (93)	0	0	0	0	0	1	1	0	1	1	1	1	1	0	1	1	1	0	10	Moderate
Roshdy et al., 2021 (115)	0	0	0	0	0	1	1	0	1	1	1	1	0	0	0	1	1	0	8	High
Farahat et al., 2022 (68)	1	0	0	0	0	1	1	0	0	1	1	0	0	0	1	1	1	1	9	Moderate
Torres-Betancourt et al., 2022 (27)	1	0	0	0	0	1	1	1	0	1	1	0	0	0	1	1	1	1	10	Moderate
Meng et al., 2020 (31)	0	0	0	0	0	1	1	1	1	1	1	1	1	0	0	1	1	0	10	Moderate
Seung et al., 2018 (117)	0	0	0	0	0	1	1	1	1	1	1	0	1	1	0	0	1	0	9	Moderate
Baras et al., 2019 (84)	1	0	0	0	0	1	1	1	1	1	1	0	1	1	0	0	1	0	10	Moderate
Liu et al., 2024 (82)	0	0	0	0	0	1	1	1	1	1	1	1	1	0	1	1	1	0	11	Moderate
Baras et al., 2019 (116)	0	0	0	0	0	1	1	1	1	1	1	0	0	1	0	1	1	0	9	Moderate
Liu et al., 2019 (37)	1	0	0	0	0	1	1	0	1	1	1	0	1	1	1	0	1	0	10	Moderate
Arias-Moliz et al., 2015 (38)	0	0	0	0	0	1	0	1	1	1	1	1	1	1	0	1	1	0	10	Moderate
Alharamlah et al., 2025 (36)	0	0	0	0	1	1	0	0	1	1	1	0	1	1	0	1	1	0	9	Moderate
Carvalho et al., 2021 (58)	0	0	0	0	0	1	1	1	1	1	1	0	0	1	1	1	1	0	10	Moderate
Kapralos et al., 2022 (50)	0	0	0	0	0	1	1	1	1	1	1	0	0	0	1	1	1	0	9	Moderate
Habib et al., 2021 (86)	1	1	0	0	0	1	1	1	1	1	1	0	0	0	0	1	1	0	10	Moderate
Mokabberi et al., 2024 (120)	0	0	0	0	0	1	1	1	1	1	1	0	1	1	0	0	1	0	9	Moderate
Ratih et al., 2023 (81)	0	0	0	0	0	1	1	0	1	1	1	0	1	1	1	0	1	0	9	Moderate
Loyola-Rodríguez et al., 2019 (63)	0	0	0	0	0	1	1	1	1	1	1	0	1	1	0	1	1	0	10	Moderate
Collares et al., 2018 (77)	0	0	0	0	0	1	1	1	1	1	1	0	1	1	0	1	1	0	10	Moderate
Kangarlou et al., 2016 (83)	0	0	0	0	0	1	1	0	0	1	1	0	1	1	0	0	1	0	7	High
Khoshbin et al., 2025 (61)	0	0	0	0	0	1	1	1	0	1	1	0	0	1	1	1	1	0	9	Moderate
Ghaffari et al., 2024 (119)	0	0	0	0	0	1	1	1	0	1	1	0	1	1	0	1	0	0	8	High
Weckwerth et al., 2015 (118)	0	0	0	0	0	1	0	1	1	1	1	0	1	0	0	0	1	0	7	High
Monteiro et al., 2019 (33)	0	0	0	0	0	1	1	0	1	1	1	0	1	1	1	1	1	0	10	Moderate
Del Carpio-Perochena et al., 2022 (122)	1	0	0	0	0	1	1	0	0	1	1	1	0	0	0	0	1	0	7	High
Rossato et al., 2017 (121)	0	0	0	0	0	1	1	1	1	1	1	0	1	1	1	1	1	0	11	Moderate

Scoring: Yes = 1; No/Unclea*r* = 0.

Total Score = 18.

Overall Risk Category: Low Risk: ≥ 14 score; Moderate Risk: 9–13 score; High Risk: < 9 score.

**Items Description.**
Randomization Reported Randomization Method Appropriate Blinding Reported Blinding Appropriate Sample Size Justified Control Group Present Negative Control Positive Control Replication Reported Statistical Analysis Appropriate Outcome Measures Clearly Defined Selective Reporting Conflict of Interest Reported Funding Source Reported Cytotoxicity Assessed Material Characterization Standardization of Methods Incomplete Data Addressed

## 4 Discussion

Root canal reinfection is a significant concern in endodontics, particularly because it is not always possible to guarantee complete disinfection of the root canal (16). As a result, various strategies have been proposed to enhance the chemo-mechanical disinfection of the root canal system, with the use of antimicrobial endodontic sealers being one of the primary approaches (7). This scoping review examines the use of different antimicrobial compounds in resin-based endodontic sealers. Resin-based sealers are the most commonly used (17), so this scoping review was designed to provide an overview of efforts to engineer them with antimicrobial and antimicrobial properties. The findings indicate that most studies conducted were *in vitro*. While assessing the efficacy of endodontic materials in more clinically relevant settings, such as living or *in situ* models, laboratory studies offer numerous valuable advantages. Laboratory research often serves as the initial stage of material evaluation, enabling faster testing and the development of new materials. By focusing on laboratory studies, unnecessary testing on humans and animals can sometimes be avoided, thereby enhancing understanding of the underlying mechanisms (18).

Both laboratory studies and other types of research are crucial. Although many variables can be controlled independently in the lab, the results of some antimicrobial sealers may not directly correlate with clinical outcomes. However, laboratory studies provide important preliminary assessments that can significantly aid the development of innovative dental materials (19). This review discusses studies utilizing microbial models as laboratory tools to evaluate the antimicrobial properties of antimicrobial sealers. While *E. faecalis* is the most tested species, it is important to emphasize that multi-species biofilms are preferred due to their greater resistance to antibiotics and antimicrobial agents. As future laboratory research aims to cultivate multi-species biofilms, this study examines a limited number of existing investigations involving materials with multi-species biofilms (20). All these studies employed static biofilm models, which do not accurately replicate the root canal environment. To address the limitations of static biofilm models, the use of localized models presents a viable alternative. Such models also permit the investigation of other host-specific factors that modulate oral biofilm formation and the associated immune response to pulpal leakage.

In this review, which underscores a significant debate regarding the selection of the optimal evaluation model, we conclude that no single model can be deemed absolutely the most suitable. The choice of model depends on various factors, including the stage of material development and research (19). For instance, simple laboratory models are typically used in the early stages of designing new materials. A chemical model may first assess the material's initial properties, followed by a microbial model. Once a material exhibits exceptional performance during laboratory evaluations, the research progresses to *in vivo* models within living organisms. This represents the most realistic environment for testing materials and evaluating their final behavior prior to clinical application.

The diversity of models employed across the reviewed studies carries important implications for the external validity of their findings. *In vitro* models, while essential for early-stage evaluation, do not replicate the complex biological environment of the root canal system, including the presence of dentinal tubules, biofilm architecture, host immune responses, and the dynamic conditions of the periapical tissues. Therefore, antibacterial or physicochemical performance demonstrated under controlled laboratory conditions may not directly translate to clinical outcomes, prioritizing the need to advance these laboratory models to more clinically representative models to better bridge the gap between laboratory evidence and clinical applicability. Furthermore, the lack of methodological standardization across studies, such as variations in bacterial strains, inoculum concentrations, contact times, and aging protocols, limits the comparability of results and makes it difficult to draw definitive conclusions about the clinical relevance of the reviewed findings.

### 4.1 Imparting antimicrobial properties in endodontic sealers using different additives

Numerous metabolites, including tannins, terpenoids, alkaloids, and flavonoids, are produced by plant extracts and represent novel antimicrobial agents against pathogens (21). Thymus Kotschyanus Boiss is among the most favored plants globally, recognized for its aromatic and therapeutic properties. Thymus essential oils and extracts are employed in pharmaceutical products due to their antimicrobial and antioxidant properties (22). In one study, *Thymus Kotschyanus Boiss* could be a useful addition to root canal sealer (23). Saha et al. assessed the effectiveness of three sealers in conjunction with three herbal extracts against seven distinct bacterial strains across various time periods utilizing the Agar Diffusion Test (24). The combination of the resin-based sealers (AH Plus®) with *Glycyrrhiza glabra* demonstrated the highest efficacy as an antibacterial agent (24).

One study used the agar diffusion method to assess the effectiveness of three sealers in addition to three herbal extracts against nine bacterial strains over various time periods (25). It was found that the combination of endodontic sealers with the extracts of Myristica fragrans, Emblica officinalis, and Emblica officinalis resulted in strong antibacterial effects (25). Bismuth lipophilic nanoparticles (BisBAL NPs) have been previously reported to exhibit exceptional antifungal activity (26). In a study by Torres-Betancourt et al. (27), AH + supplemented with BisBAL NPs has antibacterial properties by observing the growth of *E. faecalis*. When added to AH Plus, BisBAL NP was found to improve its antibacterial activity by 4.9 times compared to AH Plus alone (27).

Nano-magnesium hydroxide's (NMH) biocompatibility and wide-spectrum antibacterial activity have led to its extensive use in biomedical applications. One way to facilitate cartilage regeneration is to include NMH into synthetic polymer scaffolds (28). The healing effects of sericin on corneal lesions can be amplified by NMH (29). Furthermore, NMH was found to exhibit a non-toxic effect, in contrast to nano-silver, suggesting its significant potential for biological applications (30). In one study, the antimicrobial properties of AH PlusTM sealer containing NMH was tested against *Streptococcus mutans* biofilms (31). After 5 and 20 min of incubation with AH Plus+7% of NMH, 93.1% and 98% of *S. mutans*, respectively, were killed. When compared to AH Plus alone, the combination of 5% or 7% of NMH was more effective against *S. mutans*. (31).

Another category of antimicrobial compounds that have gained more attention recently are Quaternary ammonium compounds (QACs). QACs are well-known for their antimicrobial properties. Among different QAC, alkyl trimethyl ammonium bromide (ATAB) was found to diminish biofilm stability and serves as an efficient antibacterial agent against *E. faecalis* (32). Monteiro et al. evaluated experimental resin-based root canal sealers with halloysite nanotubes loaded with alkyl trimethyl ammonium bromide (ATAB/HNT) (33). Their findings demonstrated that the antibacterial efficacy against *E. faecalis* was enhanced with increasing concentrations of ATAB in relation to HNT percentage. In both the direct contact model and the planktonic bacteria testing, ATAB/HNT totally reduced bacterial growth.

Dimethylaminohexadecyl methacrylate (DMAHDM) and dimethylaminododecyl methacrylate (DMADDM) have shown strong antibacterial activity against several oral pathogens (34). Research has shown that resin-based materials containing DMAHDM are effective in eliminating the biofilms of many oral infections, involving *S. mutans* as well as *Candida albicans* (35). In one study, the *E. faecalis* biofilms were hindered and completely eradicated by the introduction of DMAHDM (*P* < 0.001) (36). In another study, results demonstrated that EndoREZ's antibacterial characteristics could be significantly enhanced by adding DMADDM to the sealer (37). Furthermore, in multispecies microecology, sealers containing DMADDM reduced the growth of E. faecalis (38). Another QAC that was investigated for its antibacterial properties is polyethyleneimine (QPEI) (39–42). However, when it was added to AH Plus sealer, no antibacterial effect was observed (43). A possible explanation of such findings is that the QPEI nanoparticles might position themselves in a manner that reveals their hydrophilic groups at the surface of AH Plus sealer. These groups may sustain insufficient charge, potentially because the sealer's complex chemical composition hinders with the availability of the electrical charge needed for antibacterial action.

The basis of the antimicrobial action of benzalkonium chloride was the disruption of the membrane charge distribution and alteration of the cell membrane permeability leading to the cytoplasmic components leakage, resulting in bacterial death (44). The ability of benzalkonium chloride to inhibit proteases has led to its incorporation into many dental restorative materials, involving adhesives, resin composites, glass ionomer cements, as well as concentrations as high as 5%, bestowing upon them advantageous antibacterial characteristics as well as long-lasting effects (45). Also, when incorporated into a resin composite the release of benzalkonium chloride was noted over time and the shear bond strength increases (46). The agar diffusion exam illustrate the efficacy of AH Plus was enhanced when combined with 2 wt.% of benzalkonium chloride (47). Arias-Moliz et al. investigated the physicochemical characteristics, and antibacterial activity of AH Plus sealer when combined with varying doses of benzalkonium chloride (38). They found adding BC to AH Plus greatly enhances its antibacterial effects.

In addition to its low cost and high efficiency, chlorohexidine (CHX) also exhibits substantivity, which is a significant advantage in its application (48). It is effective against fungi due to its broad-spectrum antimicrobial action (49). Kapralos et al. revealed that The antibacterial capabilities of the sealer leachates were enhanced when CHX was in contact with the surfaces of the sealers (50). Root canal sealers that contain nanoparticles (NP) have mostly been studied for their ability to improve antibacterial function and improve the substantivity of the sealers (43, 51). One of these antibacterial compounds utilized in a nanoparticulated form is CHX. NPs of CHX have newly demonstrated different materials, like silicone-based inputs (52), glass ionomer sealers (53), biomedical materials (54) and implant surfaces (55). The slow-release properties of CHX NPs combined with hexametaphosphate (CHX-HMP NPs) have been demonstrated in earlier studies (55, 56). In contrast to CHX digluconate, the solubility of CHX–HMP is lower. When employed as a coating, it has the potential to impart a constant release of CHX that lasts for three months (54). Additionally, it has the capacity to minimize the colonization of bacteria and inhibit the growth of harmful microorganisms (57). According to Carvalho et al., endodontic sealers containing CHX-HMP NPs had a greater antimicrobial effect than the control (58).

Deacetylation of chitin yields chitosan, a cationic polysaccharide with well-documented antimicrobial, biocompatible, and biofilm-inhibitory properties (59). Due to its ability to disrupt microbial cell membranes and generate reactive oxygen species, chitosan has been investigated in biomedical and dental applications, including endodontic sealers and restorative materials (60). Its favorable biological profile and antibiofilm activity make it a promising additive for enhancing the antimicrobial performance of resin-based sealers (61).

It was observed that the antibacterial efficacy of epoxy resin based (Thermseal) was greatly increased against *E. faecalis* with the addition of chitosan nanoparticles. The chitosan's antibiofilm capabilities in a model of root canal infection was also tested. After 4 weeks of biofilm growth, confocal laser scanning microscopy showed that the sealer-dentine interface of the unmodified sealers without chitosan nanoparticles had a high viable biofilm total volume, as bacterial colonization was significantly lower in samples sealed with sealers containing chitosan nanoparticles (62). There have been reports that show that when chitosan nanoparticles are added to epoxy resin-based (ERB) sealer at concentrations of 0, 10, 20, as well as 30 wt.%, a higher inhibitory zone of E. faecalis and reduced cytotoxicity were observed compared to the control (63). This mainly due to the capabilities of chitosan to generate free radicals, specifically reactive oxygen types, which can weaken the ability of bacteria to withstand stress by reducing the function of proteins and destroying deoxyribonucleic acid (64).

Silver (Ag) salts wound dressings exhibit antibacterial properties. Recently, dental resins achieved a homogeneous dispersion of silver nanoparticles (NAg) by reducing silver salts to nanoparticles (65). To explain its antibacterial activity, NAg is thought to target various locations on the bacterial cell wall. These sites are responsible for disrupting cell-wall formation, slowing cell division, and blocking DNA replication (66). To prevent detrimental impacts on the material's physical, mechanical, and color characteristics, NAg with a particle size of 2.7 nm may be used at low filler levels while still exhibiting good antibacterial qualities, in contrast to conventional macro- and micro-sized silver particles (67).

A study by Farahat et al. found that the bactericidal effectiveness of AD Seal sealer was improved when silver nanoparticles (SNP) were added (68). The release of silver ions towards microorganisms is mainly the reason why the antibacterial impact of the sealer is enhanced after introducing silver nanoparticles (69). Acting on several targets of the bacteria explains the silver antibacterial activity (70, 71). The sulfhydryl groups found in DNA and proteins are bound by silver, which unwinds DNA (72), changes the respiratory chain and hydrogen bonding, and disrupts cell wall formation and cell division (73). Bacterial cell death is occur due to increased permeability and additional destabilization of the bacterial membrane caused by silver nanoparticles (42).

### 4.2 The impact of antibacterial additives on the physical as well as sealing properties of root canal sealers

The physical features of the material, such as the setting time, may be influenced by the incorporation of antimicrobial compounds into sealers (74). This setting time is contingent upon the temperature, humidity, particle sizes, and types of components (75). The correlation of physical, antibacterial, and biological qualities can be achieved by determining the equipments' chemical composition and topography. In the study conducted by Vilela Teixeira et al., the atomic proportions of endodontic sealers were altered by adding AgVO_3_ (76). As the concentrations of AgVO_3_ grew, the percentages of silver (Ag) and vanadium (V) rose accordingly. Variations in the distribution of components on the specimens' surfaces were revealed by topography analysis. The sealers that were mixed with AH Plus's AgVO_3_ had a shorter setting time (76).

The American National Standards Institute/American Dental Association (ANSI/ADA) states that the setup time should not be over 10% of the manufacturer-specified time (75, 77). The antibacterial activity facilitated by chemicals released during the material's setting may be enhanced by a minor delay in hardening (78). Nonetheless, this duration cannot be prolonged, as the material's interaction with the periapical tissues may induce irritation and compromise biocompatibility. Furthermore, extended setting time may enhance solubility, creating niches that bacteria could inhabit, potentially resulting in reinfection (79, 80).

The antibacterial characteristics of the root canal sealer can only be achieved if it can penetrate the root canal's intricate anatomy and disinfect hard-to-reach areas without leaking into the surrounding tissues (28). The minimum flow rate for root canal sealers is 20 mm, as stated in the ISO 6876/2012 standard (77). When compared to the AH Plus control, the flow was enhanced by incorporating NAg. Since 2-(tert-Butylamino)ethyl methacrylate (TBAEMA) can increase the silver solubility by creating Ag N linkages (81), its use to dissolve silver salts may explain the enhanced flow (82). On the other hand, 5 wt.% of DMAHDM in AH Plus sealer considerably lowered the flow relative to the experimental control.

The thickness of the film is another critical feature of root canal sealers. Filling and sealing defects is the purpose of root canal sealers (83). Previous research has shown that leakage typically happens at one of three points: the sealer-gutta-percha contact, the sealer-dentin interface, or inside the sealer itself (29, 83). As a result, the core material should take up most of the canal area while the sealer is applied in a minimal volume (30, 83). Typically, the sealer-occupied areas are more prone to degradation, which is why this is the case (28). Consequently, thin layers of endodontic sealers typically give greater sealing qualities (30). It was also found that the combination of DMAHDM and NAg had no negative effect on film thickness or sealing properties, and the sealers still with a film thickness of no more than 50 m (84).

Irrigation solutions should also support sealers' biological qualities while preserving their chemical composition and physicomechanical behavior (85). Researchers found that in pure water, CHX had no effect on chemical characteristics, pH evaluation, or alkalinity of sealer leachates. However, CHX improved alkalinity after 28 days in saline water (50). BioRoot RCS kept its high alkalinity over time, in contrast to AH Plus and PCS, which showed pH values that were more neutral (86–89).

The physicochemical characteristics of AH with different concentrations of benzalkonium chloride were investigated by (38). The solubility and flow rate were tested to follow standards set by the ANSI/ADA. Additional tests were conducted to measure microhardness and contact angle. An x-ray diffraction study, together with FT-IR and attenuated total reflectance FT-IR, were used to assess the chemical alterations that occurred in the sealers. The incorporation of BC to AH with stayed continuously within the ANSI/ADA standards for endodontic cements, according to the findings of the physical testing. Adding benzalkonium chloride resulted in a substantial decrease in microhardness. The modified sealers did not exhibit any phase shifts. Finding no change to the characteristics listed in ANSI/ADA standards, they determined that AH Plus exhibited antibacterial and antibiofilm actions when BC was added at concentrations of two percent or higher (36). Sealer must be adequately flowed into the anatomical irregularities for optimum distribution. Periodontal tissues are more likely to be extruded when the flow velocity is excessive (90).

Halloysite nanotubes (HNT) doped with alkyl trimethyl ammonium bromide (ATAB) were evaluated by Monteiro et al. for their effectiveness in developing experimental endodontic sealers (33). The degree of conversion, softening ratio, radiopacity, flow, as well as film thickness were evaluated for both the experimental as well as control sealers. According to ISO 6876:2012, all groups showed flows exceeding 17 mm and film thicknesses below 50 m. Radiographic examinations successfully visualized all groups with radiopacities of at least 3 mm of aluminum. The root canal ability to provide sufficient radiopacity is essential for accurate diagnosis and for the physician to be able to see any remaining root canal system voids or porosity (91).

### 4.3 The cytotoxicity and biocompatibility of antibacterial additives in endodontic sealers

Sealers' cytotoxicity, genotoxicity, and bactericidal capabilities, among other biological features, are determined by shape, size, and ability to agglomerate, or join with other particles or materials. The antibacterial and cytotoxic effects of nanoparticles are affected by their concentration (92). Hassan et al. sought to confirm the long-term cytotoxic properties of an endodontic sealer based on epoxy resin, comparing it to AH plus alone at three time points (immediate, 2 and 4 weeks) with and without loading it with silver gold nanoparticles (93). This study is important because all materials and formulations should undergo cytotoxicity laboratory testing before being used clinically. To determine the cytotoxic activity of the various materials investigated, an *in vitro* bioassay was conducted on human tumor cell lines. An ordinary human fibroblast cell line (BJI) was used to conduct cytotoxic activities. To determine the cytotoxic activity and its potential clinical significance, an MTT assay was used (93). They demonstrated a significant disparity between the tested groups at various intervals, with elevated cytotoxic levels at 24 h and minimal values at 4 weeks.

Root canal sealers are cytotoxic to human periodontal ligament and osteoblasts to varying degrees depending on the amount of NPs they contain (94). The cytotoxicity of the sealers varies in accordance with the varied amounts of CHX-HMP NPs incorporation (58). Endodontic sealers typically exhibit a significant level of cytotoxicity immediately following manipulation (95). The individual toxicity profiles of each sealer can be better determined by comparing them to fresh ones after a long period of evaluation, therefore it would be more reliable to determine if this high cytotoxicity would persist after the initial period (95). The cytotoxicity of AH Plus was found to increase, most noticeably after 5% CHX-HMP NP incorporation (58).

Moreover, Bellis et al. (55) and Barbour et al. (53) found that CHX's cytotoxicity was reduced when it was combined with NPs. In contrast, digluconate and diacetate, two other CHX vectors, cause a rapid solubilization and distribution of CHX in the medium, leading to a large initial release. This is subsequently followed by a sudden drop or stop in release, which explains the significant cytotoxicity seen. Alternatively, CHX-HMP NPs have reduced cytotoxicity due to their limited solubility and the fact that they release CHX into the medium gradually, continuously, and over an extended period of time (53, 96).

Valle et al. found that Piper betle did not damage normal human fibroblast cells (97). To back up the claim that PB is not harmful, other investigations found varies toxicity of piper betle against murine fibroblast cell line (98), human cervical cancer cell line (99) and colon cancer cells (100). While this does not prove piper betle is fully poisonous, it does raise the possibility that it has anticancer effects. In their study, QiGhani and Mohamad looked at the antibacterial and cytotoxic effects of piper betle ethanolic extract in comparison to AH Plus sealer (101). At 48 h, piper betle added to the AH Plus sealer exhibited significantly less cytotoxicity than the control.

Ratih et al. showed that although the cytotoxic effects of the various chitosan addition doses varied in severity, all of the concentrations, including those without chitosan, were considered to be mildly cytotoxic (81). In terms of cytotoxicity, epoxy resin-based (ERB) sealer without chitosan (at a concentration of 0%) was the most harmful when tested with various concentrations of chitosan nanoparticles (63). The ERB sealer used in their investigation (AH 26) was composed of hexamethylenetetramine, formaldehyde, and epoxy resin, ingredients that were found to be poisonous and antibacterial (102, 103). In their study, cytotoxicity was observed at 24 h; however, this effect may diminish over time as formaldehyde release decreases after the seventh day (104). The solubility of the material directly influences the leaching of sealer components, which consequently affects its cytotoxic potential (105). In their investigation, it was discovered that the incorporation of 30% chitosan nanoparticles produced the least harmful effect. The addition of higher concentrations of chitosan, an inherently biocompatible material (106), to the ERB sealer may have reduced the proportion of cytotoxic components, including formaldehyde, epoxy resin, and hexamethylenetetramine, within the sealer mixture.

The lipid bilayer membrane seen in both human and bacterial cells suggests that ATAB's antibacterial activity may be associated with cytotoxicity (107). Furthermore, the cytotoxic action of QACs increases with the length of its alkyl chain (108). Because of its negative charges, ATAB interacts more easily with bacterial membranes (109). This is the Conversely, the positively charged nature of the material may contribute to its reduced cytotoxic effect on human cells (107). In contrast to the MTT tetrazolium test recommended by ISO 10993-5 (110), the Sulforhodamine B (SRB) method was used to assess cytotoxicity to human pulp fibroblasts in the study by Monteiro et al. (33). This approach has more sensitivity and less impacted by external influences.

All resin sealers showed cell survival values above 70%, and the results showed no significant variation among the groups; meeting the requirements suggested by ISO 10993-5 (110). Subsequently, the antibacterial efficacy is increased over time (111) and the risk of adverse effects on human cells is reduced when a drug carrier system, such as HNT. The antibacterial properties of ATAB and the bioactivity that can be generated by the nanotubes' silanol groups, HNT can serve as experimental resin sealers (112). Thus, incorporating ATAB/HNT into root canal sealers may represent a promising strategy for enhancing the overall efficacy of endodontic treatments.

Several limitations should be acknowledged when interpreting the findings of this scoping review. First, the included studies were predominantly *in vitro* investigations, which inherently limit the direct translation of the findings to clinical settings, as such models do not fully replicate the complex biological environment of the root canal system, including host immune responses, dentinal tubule architecture, and periapical tissue dynamics. Second, considerable methodological heterogeneity was observed across the included studies, particularly regarding bacterial strains, inoculum concentrations, biofilm maturation periods, contact times, and aging protocols, which precluded direct comparison of results and limited the ability to draw definitive conclusions regarding clinical efficacy. Third, the risk of bias assessment revealed that no included study achieved a low-risk classification, with the majority rated as moderate risk, and several as high risk, primarily due to insufficient reporting of randomization, blinding, and sample size justification. Future research employing standardized methodological protocols and clinically representative models is warranted to address these limitations and strengthen the evidence base for antimicrobial-modified resin-based endodontic sealers.

## 5 Conclusions

This scoping review provides a comprehensive overview of antimicrobial additives incorporated into resin-based endodontic sealers and their effects on antibacterial efficacy, physicochemical properties, and cytotoxicity. The findings indicate that a wide range of additives, including nanoparticles, quaternary ammonium compounds, herbal extracts, and chemical agents, can enhance antibacterial activity, particularly against Enterococcus faecalis, while generally maintaining acceptable physicochemical characteristics. Cytotoxicity profiles were largely favorable, although they varied depending on the type and concentration of additives. Despite these promising results, the evidence is predominantly derived from *in vitro* studies with considerable methodological variability, which limits direct comparison and clinical translation. Future research should prioritize standardized testing protocols, evaluation in multispecies and clinically relevant biofilm models, and validation through *in vivo* and clinical studies. These steps are essential to better establish the long-term performance and clinical applicability of antimicrobial-modified resin-based sealers.

## Data Availability

The original contributions presented in the study are included in the article/Supplementary Material, further inquiries can be directed to the corresponding author/s.
